# Diathesis

**Published:** 1909-01-16

**Authors:** 


### DIATHESIS.

When man, or some simioid ancestor, in the course of evolution developed the capacity of speech, he achieved over all other competitors in the struggle for existence an advantage the magnitude of which it is difficult indeed to overestimate: assuming, that is, that the lower animals have actually no means of communicating ideas and experiences from one to another. For he was thus enabled to transmit to his fellows, and ultimately by the invention of written or pictorial speech to his descendants, the fruits of his observations. By this means the many were able to profit by the example and precept of the cleverest, whereas formerly only the actual progeny of the exceptionally gifted individuals could participate in the fruits of their talents. But a corresponding disadvantage has modified some of the benefits of this intellectual advance: for thus arose among the multitude the habit of accepting without question the dicta of those in authority, real or assumed, a habit which has for many centuries hindered intellectual progress in a remarkable way, and is by no manner of means eradicated even now. Among the verbal formulæ which have satisfied the medical profession far too long is the term "diathesis," a hypothetical conception of vulnerability to particular diseases invoked as an explanation of their incidence. No one, of course, can fail to recognise that immunity, natural or acquired, complete or relative, to infective disorders has a very real existence. The bacteriological researches of the last two decades have established few facts so firmly. But diathesis, as the term was formerly used, hardly meant that: rather it expressed a constitutional condition to be recognised by certain gross physical characters of form and feature, especially in connection with the most dreaded of the infective diseases of this country, tuberculosis. It was that use of the word diathesis which constituted the obstacle to scientific thought and progress. A child exhibited certain physical features known collectively as "scrofula" or "struma," and was said to be of a corresponding diathesis, and as such especially vulnerable to tuberculosis. Another type, the "fine tuberculous" or "fairy" child, was also held to be in danger of the dread disease, and has frequently been the subject of elaborate description by medical authors.

Then came the many discoveries by which it was gradually established that tuberculosis is a disease so insidious that its earliest manifestations attract little or no attention. The full truth on this matter was not brought home at once, but piecemeal; and is probably not yet wholly revealed to us. Consequences of the realisation of the latency of early tuberculous infection are the various symptoms and signs now described as pretuberculous, though the phrase is a misnomer, since it is applied to what are regarded as indications of early infection. Yet a further consequence is the explanation of the old shibboleth of diathesis put forward by Dr. Leslie Mackenzie.* To him the signs of the tubercular diathesis are signs of existing tuberculosis. The fair-haired, thin-skinned fairy, equally with the thick-lipped "scrofulous" child, is, according to this view, already suffering from undiagnosed tuberculosis; and if this illuminating conception should prove well founded, it is indeed easy to see why children of these two types should so frequently afterwards display evidence of more advanced and therefore more easily demonstrable tuberculous lesions. Dr. Mackenzie points out that infants do not exhibit these "diatheses." By the time a child is old enough to be assigned to either class of susceptibles, it has already had innumerable contacts with the tubercle bacillus. He reduces the legitimate meaning of diathesis to the expression that if you take an infective fever you must first of all be capable of taking it; which, as he says, unravels no mysteries. And he compares such an explanation to that which the mediæval philosophers supplied for the soporific effects of opium, which was that the drug possessed a *virtus dormitiva*, a sleep-inducing quality. His conception of the tubercular diathesis is one that needs a good deal of working out, the result of which may conceivably be of the most profound importance.

* **"Edinburgh Medical Journal," December, 1908, p. 513.**

